# Distinct immune signature predicts progression of vestibular schwannoma and unveils a possible viral etiology

**DOI:** 10.1186/s13046-022-02473-4

**Published:** 2022-10-04

**Authors:** Moran Amit, Tongxin Xie, Frederico O. Gleber-Netto, Patrick J. Hunt, Gautam U. Mehta, Diana Bell, Deborah A. Silverman, Ismail Yaman, Yi Ye, Jared K. Burks, Gregory N. Fuller, Paul W. Gidley, Marc-Elie Nader, Shaan M. Raza, Franco DeMonte

**Affiliations:** 1 Department of Head and Neck Surgery, The University of Texas MD Anderson Cancer Center, Houston, TX USA; 2 Medical Scientist Training Program, Baylor College of Medicine, Houston, TX USA; 3 Department of Neurosurgery, The University of Texas MD Anderson Cancer Center, Houston, TX USA; 4 Division of Neurosurgery, House Ear Institute, Los Angeles, CA USA; 5 Anatomic Pathology, Head and Neck Disease Alignment Team, City of Hope Comprehensive Cancer Center, Duarte, CA USA; 6 Department of Pathology, The University of Texas M.D. Anderson Cancer Center, Houston, TX USA; 7 Department of Melanoma Medical Oncology, The University of Texas MD Anderson Cancer Center, Houston, TX USA; 8 Bluestone Center for Clinical Research, New York University College of Dentistry, New York, NY USA; 9 Department of Oral Maxillofacial Surgery, New York University College of Dentistry, New York, NY USA; 10 Department of Molecular Pathobiology, New York University College of Dentistry, New York, NY USA; 11 Department of Leukemia, The University of Texas MD Anderson Cancer Center, Houston, TX USA; 12 Brain Tumor Center, The University of Texas M.D. Anderson Cancer Center, Houston, TX USA

**Keywords:** Vestibular schwannoma, Skull base, Surgery, Immune, Viral, Progression

## Abstract

**Background:**

The management of sub-totally resected sporadic vestibular schwannoma (VS) may include observation, re-resection or irradiation. Identifying the optimal choice can be difficult due to the disease’s variable progression rate.

We aimed to define an immune signature and associated transcriptomic fingerprint characteristic of rapidly-progressing VS to elucidate the underpinnings of rapidly progressing VS and identify a prognostic model for determining rate of progression.

**Methods:**

We used multiplex immunofluorescence to characterize the immune microenvironment in 17 patients with sporadic VS treated with subtotal surgical resection alone. Transcriptomic analysis revealed differentially-expressed genes and dysregulated pathways when comparing rapidly-progressing VS to slowly or non-progressing VS.

**Results:**

Rapidly progressing VS was distinctly enriched in CD4^+^, CD8^+^, CD20^+^, and CD68^+^ immune cells. RNA data indicated the upregulation of anti-viral innate immune response and T-cell senescence. K − Top Scoring Pair analysis identified 6 pairs of immunosenescence-related genes (*CD38-KDR, CD22-STAT5A, APCS-CXCR6, MADCAM1-MPL, IL6-NFATC3,* and *CXCL2-TLR6*) that had high sensitivity (100%) and specificity (78%) for identifying rapid VS progression.

**Conclusion:**

Rapid progression of residual vestibular schwannoma following subtotal surgical resection has an underlying immune etiology that may be virally originating; and despite an abundant adaptive immune response, T-cell immunosenescence may be associated with rapid progression of VS. These findings provide a rationale for clinical trials evaluating immunotherapy in patients with rapidly progressing VS.

**Supplementary Information:**

The online version contains supplementary material available at 10.1186/s13046-022-02473-4.

## Background

Vestibular schwannomas (VS) are benign tumors arising from the vestibular division of cranial nerve VIII. The incidence of VS is 1.1 per 100,000 person-years in the United States, showing no gender preponderance [1, 2]. Advances in neuroimaging, particularly magnetic resonance imaging (MRI), have led to increasingly frequent diagnoses of smaller VS, thereby contributing to the increased incidence of VS reported in recent years [2]. Treatment options for VS include watchful waiting with serial MRI, surgical resection or radiotherapy. Given that VS tumors grow at a variable and unpredictable rate, choosing among treatment options remains difficult [3].

Surgical refinements and surgeon experience have minimized the morbidity and mortality associated with the surgical treatment of VS to 22% and 0.2%, respectively [4]. Although the goal of surgical excision is total tumor removal, adherence to the facial nerve, the brainstem, or local vasculature precludes such an extent of resection to only 29% of patients [5]. When gross total resection is achieved, the recurrence-free survival rates are 96%, 82%, 73%, and 56% at five, 10, 15 and 20 years. With a subtotal resection these recurrence-free survival rates drop to 47%, 17%, and 8% at five, 10, and 15 years [3]. Progression following subtotal resection is three times more likely than with gross total or near total resection and approximates 40% [6–9]. In such cases of subtotal resection, the increased risk of progression often necessitates further intervention, including subsequent surgical resections or radiosurgery. To date, there are no meaningful clinical or molecular markers that distinguish non-progressing or slowly progressing tumor remnants from those that will progress rapidly.

In the present study, we analyzed transcriptomes of tumors from patients with sporadic VS to identify molecular perturbations that might be used to discriminate slow-growing VS remnants from more aggressive VS remnants. The ability to identify patients whose tumor remnants are likely to progress rapidly would select these patients for multimodal treatment.

## Methods

### Patients

Between July 2005 and April 2014 tissue samples were collected during the surgical excision of tumors from 17 patients with sporadic VS. Patients with neurofibromatosis, history of radiation treatment to the head and neck region or lacking follow up data were excluded. All patients had less than total surgical excision, and all samples were collected before radiotherapy or any other adjuvant treatment. Progression was defined as any radiographic evidence of lesion growth following surgery. Progression-free survival was defined as the time from the date of surgery to the date of radiographic evidence of progression. The study was approved by our Institutional Review Board (protocol PA13-0067), and clinical samples and data were collected only after patients provided written informed consent.

### Multiplex immunofluorescence staining and image analysis

Our study cohort consisted of 8 patients with early disease progression (< 5 years after surgery), and 9 patients with either no progression or late disease progression (≥ 5 years after surgery). The 5 µm slides of the Formalin-sixed, paraffin-embedded (FFPE) tumor tissue were prepared and were stained for mIF. Multiplex immunofluorescence staining on a single slide was performed with use of the Opal 7-Color Manual IHC Kit (AKOYA Biosciences, #NEL811001KT). The antibodies used included CD4, CD8, CD20, CD68, and CD1A. The slides were scanned (Vectra Polaris, Akoya Biosciences) and the scanned images were analyzed at The University of Texas MD Anderson Cancer Center using AI-based software (VIS Image Analysis, Visiopharm).

### Sample preparation

Formalin-fixed, paraffin-embedded samples were submitted for analysis using HTG EdgeSeq panels. The area of each sample was measured, and HTG Lysis Buffer was added to obtain a per-well concentration of 6 mm^2^/35 µL. To improve sample lysis, we added proteinase K to the lysis buffer at a ratio of 1:20, and the samples were incubated at 50°C for 180 min. We added 35 µL of each sample to a single well of a 96-well plate. We also added 25 ng of human universal RNA to 3 wells to serve as a process control.

### Inform spectral unmixing

We used the Vectra Polaris 3.0.3 multispectral imaging system (Akoya Biosciences) through the full emission spectrum from 440 to 780 nm, to extract fluorescence intensity information from the images using positive tonsil controls from each run staining to calibrate the spectral image scanner protocol at 20 × magnification (0.5 µm/pixel). Each marker was quantified individually using a spectral signature for each fluorophore obtained by the “spectral unmixing library” using the same algorithm from the InForm 2.4.8 image analysis software (Akoya Biosciences). The percentages representing each marker were calculated by dividing the absolute number of each marker by the absolute number of total nucleated cells (DAPI +) on each core at each time point. Following whole-slide image acquisition, images are analyzed with inForm image analysis software to quantify the cell-level biological features. The inForm software program was developed to integrate multispectral capabilities with image analysis to (1) spectrally unmix and isolate multiple Opal signals and background autofluorescence; (2) detect different tissue architecture (e.g., tumor, stroma, vessels, and necrosis) using a machine learning–based neural network pattern recognition function; (3) segment individual cells starting with nuclei, based on DAPI, and using other markers to detect membranous and cytoplasmic regions of cells; and (4) identify cell types of interest based on marker signal levels and cellular staining pattern using user-trained multinomial logistic regression algorithms. Once the slides were stained, they were scanned on a multispectral digital slide imaging system, the Vectra Polaris. The Vectra Polaris uses multispectral imaging technology to compensate for optical spectral bleed-through among channels and to isolate signal from background autofluorescence. Using inForm, designated library slides are used to isolate the exact spectral signature of each fluorophore to properly unmix each whole-slide composite image, as well as isolate and remove tissue autofluorescence. With multispectral unmixing, residual bleed-through was reduced to < 1% in both cases.

### HTG EdgeSeq assay

Samples were run on an HTG EdgeSeq processor using an HTG EdgeSeq Oncology Biomarker Panel that allows for the measurement of expression of 2,549 genes (Supplementary Table S1). The samples were then individually barcoded using a 19-cycle PCR reaction to add adapters and molecular barcodes. Barcoded samples were individually purified using AMPure XP beads and quantitated using a KAPA Library Quantification kit. The library was sequenced on an Illumina MiSeq using a V3 150-cycle kit with 2 index reads. PhiX was spiked into the library at 5%; this spike-in control is standard for Illumina sequencing libraries.

Data were returned from the sequencer in the form of de-multiplexed FASTQ files, with one file per original well of the assay. To collate the data, we used HTG EdgeSeq Reveal software to align the FASTQ files to the probe list. We applied Median Ratio Normalization (MRN) to the aligned raw data prior downstream analysis. MRN data is available in the Supplementary Table S2.

### Identification of differentially expressed genes

Gene expression analyses were performed using JMP PRO 15.2.1 software. To identify differentially expressed genes between patients with early (< 5 years) and late (≥ 5 years) progression, we performed unpaired two-sample Wilcoxon test and the *p*-value correction for multiple testing was done by the Benjamini & Hochberg FDR method. However, considering the small sample size (*n* = 17), we considered unadjusted *p*-values for downstream analyses. Gene expression patterns among samples were visualized by hierarchical clustering analysis (HCA) using the Ward minimum variance method for defining distances between clusters, and by principal component analyses (PCA).

### Canonical pathway integrative analysis

To determine the potential biological mechanisms associated with the gene expression differences between the groups of schwannoma samples, we initially performed an overrepresentation analysis (ORA) using the KOBAS 3.0 online tool (http://kobas.cbi.pku.edu.cn) [10]. This software evaluates whether a list of genes is statistically enriched by pathways and terms from several databases such as KEGG, Reactome, and Gene Ontology (GO). The gene list used for this analysis was performed by differentially expressed genes (*p* < 0.01) between early and late/no progression VS cases. ORA results were considered significant at FDR < 0.05 (by Benjamani and Hochberg).

To further explore the biology behind the transcriptional differences between the VS groups, we used the Ingenuity Pathway Analysis (IPA) software (Qiagen) that indicates whether differentially expressed genes are involved in the activation or inhibition of a curated set of canonical pathways and other biological mechanisms. IPA uses a priori knowledge of expected interactions between transcriptional regulators and their target genes stored in the Ingenuity Knowledge Base, a scientific literature–based database. Considering that the IPA analysis is more complex and considers statistical parameters associated with the degree of transcriptional variation between the compared groups, all differentially expressed genes (*p* < 0.05) between VS groups were used as input. Pathways were considered significantly enriched when the z-score was < 1 or > 1 and *p* < 0.05. Positive z-scores are indicative of activation, while negative z-scores are suggestive of inhibition of a given pathway.

Additionally, we investigated the existence of protein–protein interaction evidence among genes differentially expressed between VS groups (*p* < 0.05) using the online tool STRING. Gene interactions were considered only at the highest confidence score (> 0.9) [11]. After filtering out genes without significant interactions, an additional ORA was performed using the STRING database.

### Prediction model development

To evaluate potential predictive markers to discriminate between patients with early progression and those with late or no progression, we employed the k − Top Scoring Pair (KTSP) classifier [12]. Briefly, KTSP is based on the Top Scoring Pair approach proposed by Geman et al. [13], in which gene expression data are converted into binary classifiers based on the expression level difference between 2 genes (e.g., *GeneA* > *GeneB* and *GeneA* < *GeneB*). The best gene-pair classifiers are those whose expression levels switch more consistently between the 2 groups of interest (e.g., *GeneA* > *GeneB* in 90% of group 1 samples, whereas *GeneB* > *GeneA* in 100% of group 2 samples). The KTSP approach is based on the same concept but considers a “k” number of gene pairs in the final prediction algorithm. KTSP initially calculates the discriminatory power—called “votes”—for all possible gene pairs in a gene expression set. The prognostic score is obtained by summing the votes among all “k” pairs. The best prognostic marker is that for which the “k” number of pairs provides the highest score [12]. KTSP calculations were performed with the switchBox package in R [14].

### CD8+ T cell isolation, activation, and co-culture with cancer cells (TR6Bc1), and single cell immune analysis

Mouse schwannoma cell line, TR6Bc1, is maintained in our lab with DMEM medium (with additional 2 mM Glutamine, 10% tryptose phosphate broth, and 10% fetal bovine serum) and was plated into a cell culture insert (24 well format) 24 h before co-culture with T cells. CD8^+^ T cells were then isolated and activated as previously described [15]. Briefly, spleen was harvested from C3H mice and was pressed though cell strainer. Red blood cells (RBC) were lysed with lysis buffer (eBioscience 10X RBC Lysis Buffer (Multi-species), ThermoFisher). The CD8^+^ T cells were isolated with CD8^+^ T Cell Isolation Kit (Miltenyi Biotec B.V. & Co. KG) following the company’s protocol and activated for 18 h with CD3 antibody (coated, 2 µL/mL) and CD28 antibody (5 µg/ml) (BioLegend) in RPMI-1640 medium. After activation, the T cells were plated in a 24 well-plate and cultured with or without TR6Bc1 cells, for 24 h. Next, the T cells were harvested and stained with Cell stain 405 and anti-CD8 antibody (Alexa Fluor 647 anti-mouse CD8a (AF647-CD8) (IsoCode Kit, Isoplexis) following the manufacturer’s protocol. After staining, the T cells were re-suspended in RPMI-1640 medium (1 × 10^6^ cells/mL) and 30 µL of the T cells was transferred into the inlet port of the IsoCode chips (IsoCode Kit, Isoplexis). The chips were then loaded into the Isolight instrument. The data for single cell CD8^+^ immune function were analyzed with IsoSpeak Software (Isoplexis).

## Results

### Rapidly progressing and slowly/non-progressing VS have distinct immune signatures

**Fig. 1:**
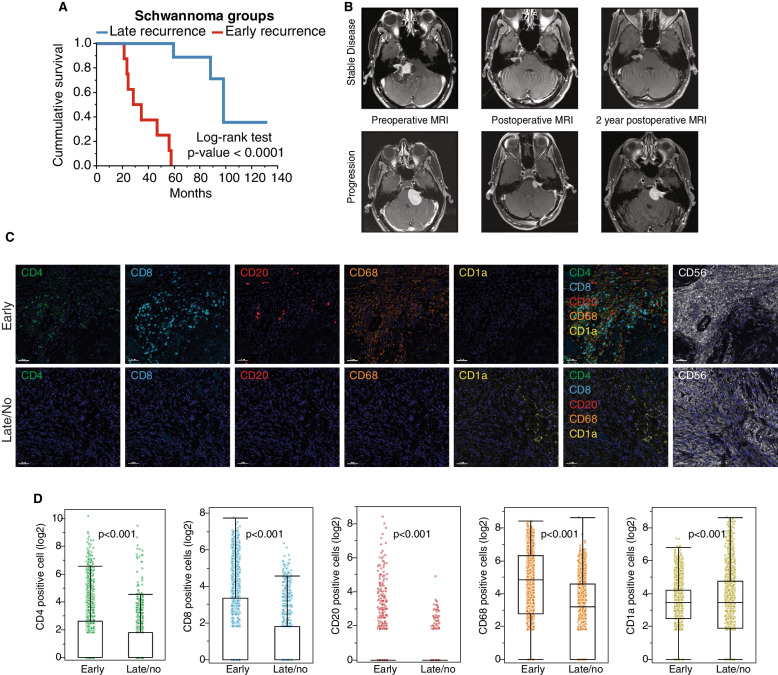
Increase immune cell infiltration is associated with rapid progression of vestibular schwannoma (VS). **A** Kaplan–Meier analysis showed that the time to recurrence for VS patients with early recurrence (*n* = 8; red line) was significantly shorter than that for patients with late recurrence (*n* = 9; blue line; *P* < 0.0001, log-rank test). **B** Axial T1-weighted, postcontrast magnetic resonance imaging at the level of the cerebellopontine angle demonstrates the pre-, early post-, and late postoperative statuses of a patient with stable VS (top row) and a patient with rapidly progressing VS (bottom row). **C** Representative multiplex immunofluorescence imaging of immune markers (CD1A, CD4, CD8, CD20, and CD68) and tumor markers (neural cell adhesion molecule/CD56) in VS samples from patients with early recurrence (top row) and late recurrence (bottom row). **D** Distributions of cells positive for CD4 (green), CD8 (blue), CD20 (red), CD68 (orange), and CD1A (yellow) among patients with early (E) and late (L) progression. Cell density was calculated as the number of cells per square millimeter and was log_2_-transformed for representation. Compared with samples from patients with late recurrence, those from patients with early recurrence had significantly higher densities of cells positive for CD4 (67.1 ± 571 vs. 32.9 ± 395.8 cells/mm^2^), CD8 (50.9 ± 148.1 vs. 9.54 ± 31.7 cells/mm^2^), CD20 (8.16 ± 112.1 vs. 0.81 ± 4.64 cells/mm^2^), and CD68 (384.8 ± 563.0 vs. 90.8 ± 206.8 cells/mm^2^) but a significantly lower density of cells positive for CD1A (60.7 ± 99.5 vs. 186.9 ± 526 cells/mm.^2^; *P* < 0.001 for all, Wilcoxon test)

Our study cohort consisted of age- and sex-matched patients with VS treated with surgery alone at The University of Texas MD Anderson Cancer Center from 2005–2014 stratified by those with early disease progression (< 5 years after surgery; *n* = 8) or either no progression or late disease progression (≥ 5 years after surgery; *n* = 9; Fig. 1A, B, Table 1). From patient tumors, we identified 5 major intratumoral immune cell types: CD4^+^ T cells, CD8^+^ T cells, CD20^+^ B cells, CD68^+^ macrophages, and CD1A^+^ dendritic cells. Using inForm spectral unmixing, we classified the phenotypes of 3.3 × 10^5^ immune cells within neural cell adhesion molecule-positive tumor regions. Quantification of the innate immune cells revealed a significant enrichment of CD68^+^ macrophages and a significant depletion of dendritic cells (CD1a^+^) in rapidly progressing VS (*P* < 0.001; Fig. 1C, D). Regarding the adaptive immune cells, there was a significant enrichment in CD4^+^ and CD8^+^ T cells in rapidly progressing VS (*P* < 0.001; Fig. 1C, D). Although the overall number of CD20^+^ B cells across the tissue samples was low, a spectral isolation algorithm allowed us to confidently identify their phenotypes despite the low expression of CD20 across the tissue samples; this analysis revealed that the distribution of CD20^+^ B cells differed significantly between rapidly progressing VS and slowly progressing VS (*P* = 0.008; Fig. 1C, D). These different cellular distributions suggest that rapidly progressing VS and slowly progressing VS have distinct immune signatures in both the innate and adaptive immune compartments.

**Table 1:** Patients’ demographic and disease characteristics by recurrence time

Characteristic	Early recurrence, *n* = 8	Late recurrence, *n* = 9	*P*-value
Sex			> 0.99^a^
Male	3	3	
Female	5	6	
Age, years			0.58^b^
Mean ± SD	52.8 ± 12.6	49.8 ± 9.8	
Median	54.9	52.2	
Tumor location			0.56^c^
Cerebellopontine angle	3	3	
Extension into the auditory canal	4	5	
Intracanalicular extension	1	0	
Extension compressing the pons & midbrain	0	1	
CN involvement			0.20^a^
CN V	6	2	
CN VII	5	7	
Mean tumor size at presentation ± SD, mm	26.9 ± 6.3	20.4 ± 11.0	0.17^b^
Presenting symptom			0.41^c^
Facial numbness	6	2	
Hearing loss	4	9	
Tinnitus	2	4	
Aural fullness	2	2	
Vertigo	2	1	
Imbalance	1	2	
Headache	1	2	
Taste changes	2	0	
Cheek numbness	1	0	
Trigeminal neuralgia	1	0	
Ataxia	0	1	
Facial weakness	0	1	
Hyperacusis	0	1	
Mean follow-up duration ± SD, years	7.8 ± 2.6	8.6 ± 2.2	0.51

All data are no. of patients unless otherwise indicated

*SD* Standard deviation, *CN* Cranial nerve

^a^Calculated using Fisher exact test

^b^Calculated using t-test

^c^Calculated using chi-square test

### Innate immune response pathways are enriched in rapidly progressing VS

**Fig. 2:**
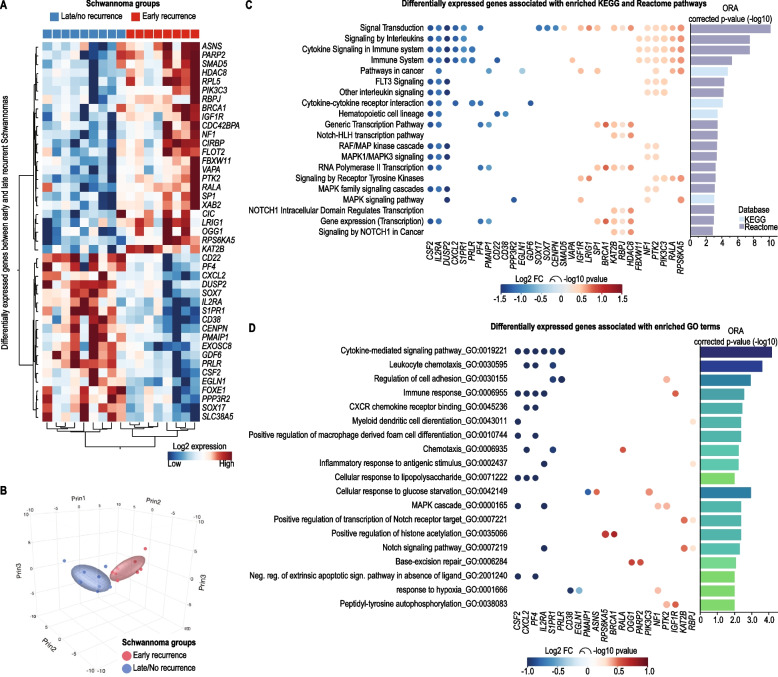
Rapidly progressing vestibular schwannoma has an enrichment of genes associated with innate immune cell activation. **A** Heatmap showing 44 genes differentially expressed (*p* < 0.01) between vestibular schwannoma patients with early (red) and late (blue) recurrence. Gene expression levels are represented in log_2_ scale. **B** Principal component analysis with the same set of 44 genes confirmed that tumors from patients with early recurrence and those from patients with late recurrence have distinct gene expression profiles. **C** The Bubble plot shows KEGG and Reactome pathways significantly enriched by the 44 differentially expressed genes (DEG) between the early- and late-recurrence VS groups (left y-axis). Enrichment corrected *p*-values were -log_10_ − transformed for representation (right y-axis). Each DEG associated with the depicted enriched pathways are represented by a bubble, in which their color and size represents the gene log_2_ fold-change and -log_10_ transformed *p*-values obtained from VS group comparison. **D** The Bubble plot shows Gene Ontology (GO) terms significantly enriched by the 44 differentially expressed genes (DEG) between the early- and late-recurrence VS groups (left y-axis). Enrichment corrected *p*-values were -log_10_ − transformed for representation (right y-axis). Each DEG associated with the depicted enriched pathways are represented by a bubble, in which their color and size represents the gene log2 fold-change and -log10 transformed *p*-values obtained from VS group comparison

To interrogate the etiology of rapidly-progressing VS immune cell enrichment, we performed gene expression analyses using HTG EdgeSeq next-generation RNA sequencing. We analyzed the expression of 2,549 genes in 17 samples from the same patient cohort. Rapidly progressing VS samples exhibited 19 downregulated, and 24 upregulated genes, when compared with slowly or non-progressing VS cases (Fig. 2A, B) (Supplementary Table S3). To investigate the functional roles of these differentially expressed genes, we performed overrepresentation analyses (ORA) against several molecular databases (Supplementary Table S4). Considering the results obtained from the KEGG and Reactome databases (Fig. 1C), the differentially expressed genes were involved in immune activity and signal transduction, especially with Ras-ERK and PI3K-mTOR and their downstream signaling pathways. Similar findings were observed considering the enriched terms from the GO database (Fig. 2D). Genes such as *NF1* (Neurofibromatosis type 1), *RALA* (RAS Like Proto-Oncogene A), *PIK3C3*, and *RPS6KA5*, upregulated in early VS progressors, were linked to the enrichment of most of the Ras-ERK and PI3K-mTOR cell signaling pathways and their downstream targets. On the other hand, the cytokines *CSF2* and *CXCL2* and the cytokine receptor *IL2RA*, were downregulated in early VS progressors, and, among other downregulated genes, were enriched among immune-related pathways and GO terms. The enrichment of pathways associated with immunity corroborates with our microscopic findings. Additionally, it suggests that a gamut of cell signaling pathways are altered among VS cases, which could represent a potential mechanism for our findings.

**Fig. 3:**
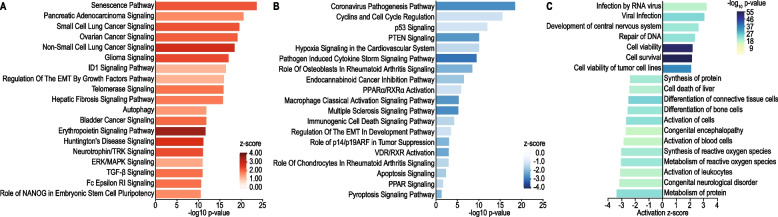
Pathways significantly enriched in rapidly progressing vestibular schwannoma. **A** Ingenuity Pathway Analysis (IPA) canonical pathways predicted to be activated (positive z-score) in early recurrent VS patients. The predicted level of pathway activation (z-score) is represented by bar color and its activation significance level (-log_10_ transformed *p*-value) by bar length (y-axis). **B** IPA canonical pathways predicted to be inhibited (negative z-score) in early-recurrent VS patients. The predicted level of pathway inhibition (z-score) is represented by bar color and its activation significance level (-log_10_ transformed *p*-value) by bar length (y-axis). **C** IPA Disease & Function terms predicted as significantly activated (positive z-score; x-axis) and inhibited (negative z-score; x-axis) among early recurrent VS patients. Significance levels are represented by bar color (-log_10_ transformed *p*-value)

To improve our perspective on the direction of molecular enrichment changes between the VS groups, we explored our findings using the IPA software that provides an estimated activation or inhibition score (z-score) for significantly enriched pathways. Cellular senescence, cancer, epithelial-mesenchymal transition, and Ras-ERK & PI3K-mTOR-related signaling, were the top enriched pathways displaying a positive z-score (Fig. 3A), suggesting that these pathways are activated among fast-progressing VS. On the other hand, immune, stress response, and tumor suppressor signaling pathways were found significantly inhibited among fast VS progressors. These results corroborate with our other findings, suggesting that early VS progressors have a decreased local immunity associated with immune signaling inhibition. Additionally, this phenotype is accompanied by an increase of signaling pathways associated with malignant neoplasms, especially those pathways directly or indirectly related to Ras-ERK & PI3K-mTOR signaling, which are major modulators of survival and proliferation mechanisms.

### Altered adaptive immune response is associated with a possible viral etiology in rapidly progressing VS

The lack of enrichment in adaptive immune response − related pathways in the presence of increased B- and T-cell populations suggests adaptive immune exhaustion or dysfunction in rapidly progressing VS. When taken in the context of well-preserved, upregulated innate immunity − related pathways in the presence of severely altered adaptive immune response, we hypothesized that adaptive immunosenescence underlies rapidly progressing VS. This hypothesis is supported by the identification of neuroinflammatory and cellular senescence pathways enriched in rapidly progressing VS (Fig. 3A, B and Supplementary Tables S5 and S6).

Although immunosenescence is most often associated with aging, it can also result from continuous exposure to external perturbations. In fact, we observed an enrichment of several stress-related pathways upregulated in rapidly progressing VS, such as Senescence, Telomerase Signaling, Hepatic Fibrosis Signaling Pathway, Autophagy, LPS-stimulated MAPK Signaling, Role of BRCA1 in DNA Damage Response, Activation of IRF by Cytosolic Pattern Recognition Receptors, UVB-Induced MAPK Signaling, among others, suggesting that these tumors are under the effect of a stressor. Interestingly, we found that rapidly progressing VS cases exhibited activation of several pathways linked to viral infection, such as the canonical pathway NF-κB Activation by Viruses, and others highlighted in Fig. 3C. These findings may indicate that fast progressing VS are associated with an underlying viral infection, which is associated with the dramatic upregulation of stress-related and proliferation/survival signaling pathways, as well as the significant downregulation of immune signaling.

**Fig. 4:**
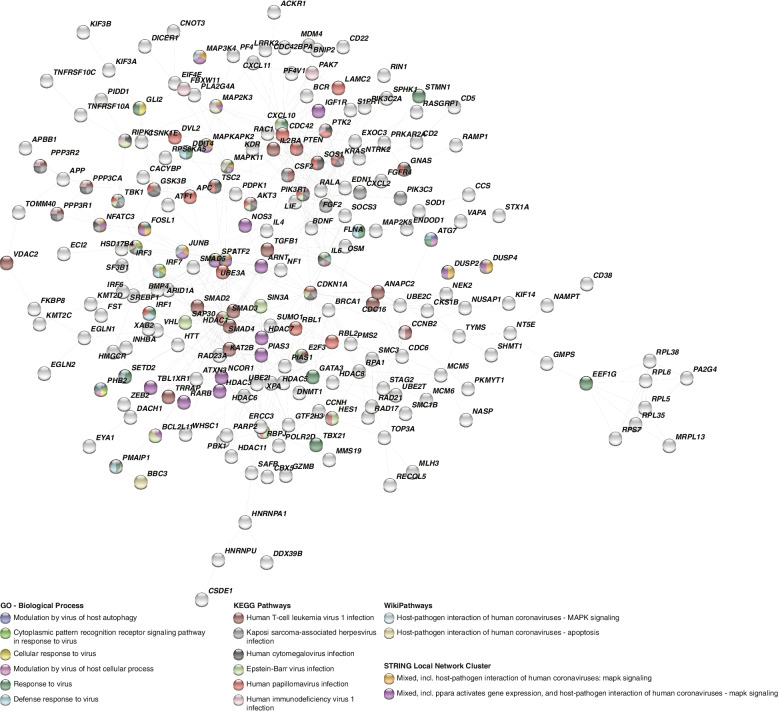
Protein–protein interaction analysis identifies gene hubs associated with viral infection-related pathways in rapidly progressing vestibular schwannoma. The figure depicts the protein–protein interactions with high level of confidence (gray lines) that are established among genes (circles) differentially expressed between VS groups (*p* < 0.05). Circle colors indicate whether a gene is known to be associated with a specific viral-related pathway or not (grey circles)

We used the STRING database to construct a protein–protein interaction network. The protein–protein interaction network consisted of 343 nodes and 559 edges with a significant number of interactions (*P* < 0.0001). Considering only the genes with known high confidence interactions with each other, we performed an additional ORA using the STRING database. These genes significantly enriched several pathways and GO terms associated with molecular signaling associated with viral infection, especially mechanisms associated with immune innate response against viruses (Fig. 4, Supplementary Table S7).

### Immunosenescence gene signature predicts rapid VS progression

Given our finding that rapidly progressing VS has enriched expression of immune response-related genes, we focused our biomarker discovery approach on 438 immune-related genes included in our HTG EdgeSeq Oncology Biomarker panel (designated “Cluster of Differentiation” and “Immuno-Oncology”). k − Top Scoring Pair (KTSP) analysis identified 9 gene pairs (18 genes total) whose combined scores provided the best discriminatory power between rapidly progressing VS and slowly or non-progressing VS. The finding of more CD4^+^ and CD8^+^ T cells in rapidly progressing VS in the absence of increased T-cell activation levels suggests immunosenescence.

**Fig. 5:**
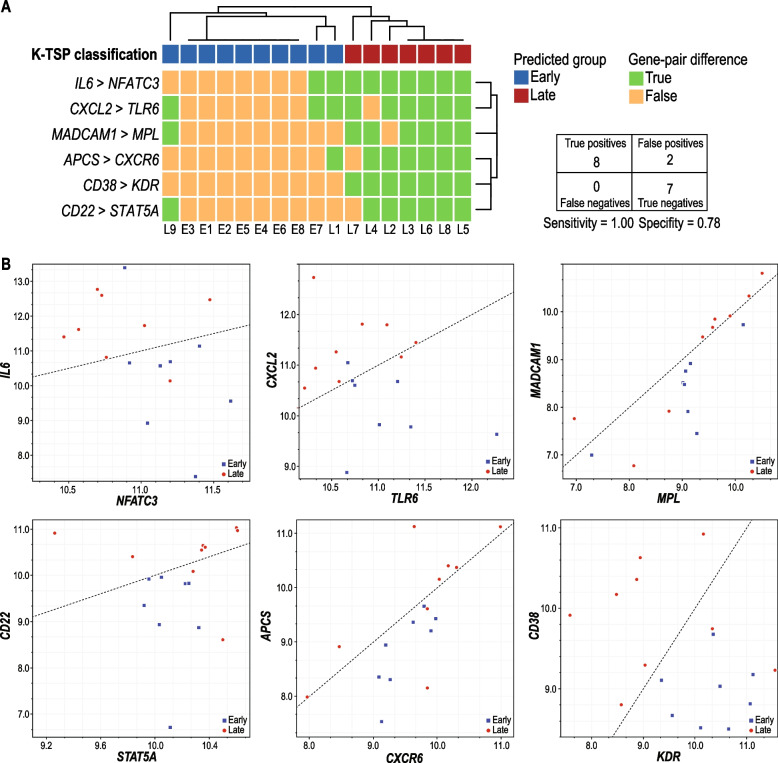
Relative expression of immune-related genes is associated with outcomes of vestibular schwannoma (VS). **A** Expression differences in pairs of genes are represented in green (“True”) if the expression of the first gene was higher than that of the second gene or in orange (“False”) otherwise. KTSP classification (top row) was calculated by summing votes for each gene pair; red indicates predicted late recurrence, and blue indicates predicted early recurrence. The bottom row indicates the correct group for each sample; L indicates late recurrence, and E indicates early recurrence. The KTSP model correctly categorized all 8 of the patients with early recurrence and 7 of the 9 patients with late recurrence. **B** The scatter plots show the expression levels of each gene pair (individual genes in each pair are indicated on the y- and x-axes, respectively) in each of the 17 VS patients. The black line represents the gene-pair classification boundaries; samples represented above the line have higher expression of the y-axis gene than the x-axis gene and are classified as “True,” and samples below the line have higher expression of the x-axis gene than the y-axis gene and are classified as “False.” Blue triangles and red circles indicate samples from patients with early VS recurrence and those with late VS recurrence, respectively

Immunosenescence is most often described as a state in which either the number or function of a patient’s total T cells is markedly poorer than those of younger or healthier patients. This decrease in available or functional T cells is thought to mediate older individuals’ increased susceptibility to disease [16]. Hence, we further pruned the list of 18 genes based on the impact of these genes’ relative up- or downregulation on immunosenescence. KTSP analysis of the final list of 12 genes revealed several genes that correlate with functional adaptive immune activity—and thus negatively correlate with immunosenescence—that were upregulated in our samples. These genes included *CD38*, *CD22*, *MADCAM-1*, *APCS*, *IL-6*, and *CXCL2*. Conversely, our analysis uncovered several genes associated with T-lymphocyte dysfunction that were downregulated. The downregulation of these genes, which included *KDR*, *CXCR6*, *STAT5A*, *MPL*, *NFATC3*, and *TLR6*, also signifies a healthy adaptive T-cell response. The overexpression of each of these genes typically results in the impaired T-cell activity seen in immunosenescence. This final analysis generated a prognostic gene-set biomarker composed of 6 gene pairs (*CD38-KDR*, *CD22-STAT5A*, *APCS-CXCR6*, *MADCAM1-MPL*, *IL6-NFATC3*, and *CXCL2-TLR6;* Fig. 5A, B), which correctly included all rapidly progressing VS in our cohort and correctly excluded 7 of 9 slowly or non-progressing VS (sensitivity = 1.00, specificity = 0.78; Fig. 5A, B). of note, the two patients that were incorrectly classified as rapidly progressing VS, had relatively large tumors (i.e. 28 and 30 mm); yet neither experienced disease progression during follow up period.

### Single cell proteomic analysis shows loss of polyfunctionality in CD8+ T cells cultured with VS cells

**Fig. 6:**
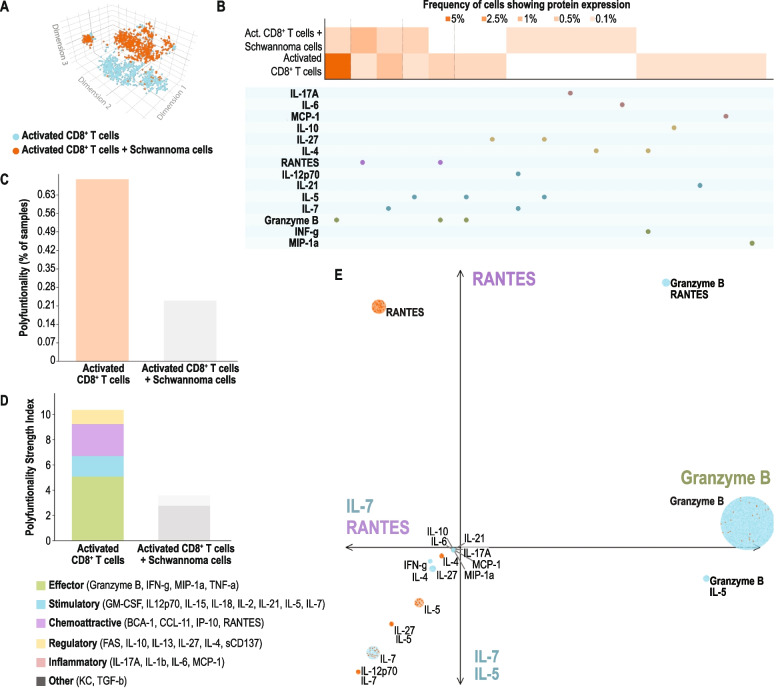
Decreased adaptive immune response in activated CD8^+^ T cells co-cultured with schwannoma cells. **A** Principal Component Analysis of a population of activated CD8^+^ T cells with (orange) and without (blue) co-cultured schwannoma cells. **B** Heat map demonstrating the difference in proteomic profile expression in activated CD8^+^ T cells with and without co-cultured schwannoma cells. **C** Cumulative polyfunctionality metric of activated CD8^+^ T cells with and without co-cultured schwannoma cells. **D** Single marker expression distribution in activated CD8^+^ T cells cultured alone (blue) or co-cultured with VS cells (orange). **E** Activated CD8^+^ T cell functional subset abundance in the presence or absence of VS cells. Note that the CD8^+^ T cells cultured with VS lost expression of their functional markers (grey)

To test our hypothesis that VS can alter immune function leading to suppressed immunosurveillance that potentially allows for VS progression, we next sought to characterize the functional impact of VS on effector T cell function. To accomplish this, we analyzed single cell proteomic profiles (Mouse Adaptive Immune panel, IsoPlexis, USA) of activated murine T cells cultured with and without murine vestibular schwannoma TR6Bc1 cells. Principal component analysis showed that CD8^+^ T cells cultured with vestibular schwannoma cells are functionally distinct from activated T cells cultured alone (Fig. 6A – C). In the presence of VS cells we found a significant downregulation of effector phenotype markers (e.g. Granzyme B, IFNγ and TNFα), chemoattractive molecules (e.g. IP-10, CCL11 and RANTES), inflammatory markers (e.g. IL17A and MCP1) and stimulatory signals (e.g. GM-CSF, IL2 and IL7) (Fig. 6D – E). Taken together, the loss of effector T cells polyfunctionality in the presence of VS supports the notion that schwannoma cells might drive immunosenescence in the tumor microenvironment of patients with rapidly progressing VS.

## Discussion

VS recur and progress at unpredictable rates, complicating disease management especially in the context of less than total surgical resection. Our results shed light on the potential mechanisms involved in VS progression. We found that multiple pathways, particularly immune-associated pathways involved in innate immunity and antigen presentation, are dysregulated in rapidly progressing forms of sporadic VS. Surprisingly, many of the cell replication- and tumorigenesis-associated pathways were not enriched, and some of these pathways (e.g., those related to DNA synthesis and central nervous system solid tumor formation) were significantly downregulated in patients with rapidly progressing VS. Taken together, these results indicate that the drivers of VS progression are external rather than internal genetic perturbations.

The drivers of sporadic VS associated with progression have never been characterized previously, although electron microscopical studies of bovine schwannomas have revealed viral particles in the tumors, suggesting a potential viral etiology [17, 18]. In the present study, we uncovered the involvement of viral response pathways, underscoring the importance of adaptive immune dysfunction in rapidly progressing VS, and perhaps even a role of viruses themselves in VS oncogenesis. Our findings show that in rapidly progressing VS, virus-inducible cellular gene networks, such as interferon gamma signaling, are enriched. The timing of potential viral infection still remains to be elucidated, that is, whether viral infection occurred after initial surgery for VS or whether the presence of such enrichment represents a reactivation of latent viral infection [19]. Yet the interaction between a virus and its host organism, which is innately inflammatory, can modify the tumor immunobiology, and may overwhelm and inhibit specific acquired responses necessary to achieve viral clearance. Similarly, we detected significant dysregulation of the expression of Toll-like receptors and IRF3 and IRF7 in sporadic VS. Upon activation, Toll-like receptors recruit adapter proteins that orchestrate inflammatory responses by the infected cells; immune cells in the tumor microenvironment that have detected a virus may also release anti-viral factors such as interferons.

Our findings showed a recruitment of CD4^+^, CD8^+^, and CD20^+^ cells to the tumor microenvironment in rapidly progressing VS. However, RNA sequencing suggested that these lymphocytes are senescent. Hence, we evaluated the predictive role of these cells’ dysregulated immunosenescence-related genes; these genes, identified and validated in our cohort, comprise the prognostic biomarker for VS progression rate. Our final biomarker is composed of 6 gene pairs, each known to play a distinct and important role in adaptive immune function: *CD38-KDR*, *CD22-STAT5A*, *APCS-CXCR6*, *MADCAM1-MPL*, *IL6-NFATC3*, and *CXCL2-TLR6.* CD38, expressed on activated T-cells is a well-defined surface protein denoting a robust T-cell response [20]. Other identified genes, are also indicative of a healthy T-cell response, although they are expressed on cooperating immune cells [21]. CD22, expressed on activated B-cells, denotes T-cell:B-cell crosstalk [22]. MADCAM-1 is a mucosal adhesion molecule that binds surface proteins on T lymphocytes, thereby guiding them into lymphoid tissue for activation [23]. Although MADCAM-1 has been studied primarily in the context of gastrointestinal lymphoid tissue [24], queries of the GTEx database reveal robust MADCAM-1 expression in several other organs, including the thyroid and brain. In line with these findings, our data indicates that MADCAM-1 expression was increased in late recurring VS.

Other genes associated with T-cell activity, like *APCS*, do not directly interact with T lymphocytes. Rather, they interact with important T cell-related proteins, thereby indirectly reflecting T-cell activity. APCS, also known as SAP, is a major acute-phase reactant protein that is dramatically upregulated during IL-6-mediated inflammation [25, 26], indirectly driving T-cells to release further IL-6, IL-17, and CXCL2. Thus, the increased expression of APCS, IL-6, and CXCL2 found in our patient cohort corresponds with improved immune cell function, and subsequent delays in VS tumor recurrence. Additionally, the 6 markers (*KDR*, *CXCR6*, *STAT5A*, *MPL*, *NFATC3*, and T*LR6)* we found to be downregulated in our late-recurring tumor samples are also biologically relevant to functional T-cell activity, which helps explain their expression and function in this context.

Other identified biomarker genes impact T-cell migration and differentiation rather than cytotoxicity and activation. Activated KDR, also known as VEGFR2, acts in an immunosuppressive manner by inhibiting the migration of T lymphocytes [27, 28]. Concordantly, the inhibition of KDR activity with an anti-KDR antibody improves the T-cell response [29, 30]. Thus, the decreased expression of KDR seen in our analysis associates with a stronger immune response and a subsequent delay in tumor recurrence. Another membrane-bound marker of T-cell activity is CXCR6, a chemokine receptor expressed on T lymphocytes that is downregulated upon T-cell activation [31]. Thus, the downregulation of CXCR6 expression is a useful biomarker of functional T-cell response. In our patients, downregulation of CXCR6 associated with better tumor control and late VS recurrence. In concert with this, CXCR6 knockout mice are better able to control infections that are known to depend on a T-cell response. However, these effects appear to be independent of T-cell activity, suggesting the need for further work to elucidate the mechanism underlying these effects [32].

STAT5A is a multi-functional protein that regulates several immune-related processes, including T-cell differentiation [33, 34]. However, while serving as a potential marker of adaptive immune function, STAT5A activity and overexpression predict both early recurrence and tumor aggression in head and neck carcinoma [35], as well as prostate cancer patients [36], matching our findings in VS. Similarly, the protein MPL, also known as CD110, is vital for proper immune function; MPL knockout mice generate only 10% of the megakaryocytes that wild type mice do [37]. However, MPL overexpression is associated with increased aggression and poor prognosis in cancer patients [38], again mirroring the expression pattern and cancer phenotype seen in our cohort. Given these proteins’ relation to proper immune cell function and the importance of immunosenescence in cancer development, progression, and recurrence, the misregulation of STAT5A and MPL likely mediates increased oncogenesis in VS via their immune-related pathways.

Nuclear factor of activated T cells 3 (NFATC3) is a transcription factor that promotes the expression of several genes that are essential for proper T-cell development and activity. Knockout studies of members of the NFAT gene family demonstrate a functional redundancy between family members [39]. Thus, NFATC3 downregulation may not be particularly damaging to a cell. However, NFATC3 also drives the expression of stem cell-promoting proteins like OCT4 [40]. Thus, NFATC3 overexpression and hyperactivity may be particularly oncogenic. Similarly, TLR6 is a membrane-bound receptor found on the surface of T cells. TLR6 activation, a sign of innate immune activation, results in inflammation at the site of expression [41]. Inflammation is a key immune-related factor in cancer development and progression, and sequence variation in TLR6 predicts inflammation-related cancer development [42]. Moreover, TLR6 overexpression is associated with poor outcomes in patients with certain cancers [43]. These descriptions match the findings of the present study, in which NFATC3 and TLR6 overexpression were associated with early recurrence, whereas decreased NFATC3 and TLR6 expression were associated with late recurrence. Given the links between NFATC3 and TLR6 activity and proper immune system functioning, the overexpression described here likely has both immunosenescent and oncogenic effects. Therefore, similar to the decreased expression of KDR, CXCR6, STAT5A, and MPL, the decreased expression of NFATC3 and TLR6 is an indicator of functional T-cell activity and subsequent late VS recurrence.

In vitro, VS cells induce “deactivation” of CD8^+^ T cells; these findings might indicate that inhibited immune surveillance results in rapid progression of VS and raise the tantalizing possibility that drugs that target immune cell regulation could be useful in the treatment of VS.

Our study was limited by the low number of patients. Additionally, this is the first analysis of its kind in sporadic VS, and there currently exist no external datasets with available expression data; hence, our work requires further validation, including validation using in vivo models. Still, our well-defined, surgically-treated patient population enabled the detection of signals that allowed us to further explore the possibility of a viral link with progression of residual tumor following the subtotal resection of sporadic VS. Our data confirm the role of innate immune response in, and the potential viral etiology of, VS progression. Our study tested a relatively small number of genes (2,549) and it employed bulk rather than single-cell sequencing. Although the assay we used was not designed for the evaluation of the impact that external perturbations have on the tumor microenvironment, its results clearly demonstrate their role in disease progression. These novel results provide data that support further investigation of the immunobiology of VS. Further studies are needed to identify any potential viral pathogens, prior to considering any antiviral approach. The senescent or exhausted adaptive immune microenvironment in rapidly progressing VS, however, suggests a potential role for immune checkpoint (e.g. anti-PD1) and innate immune cell targeting.

## Conclusion

The rapid progression of residual VS following subtotal surgical resection has an underlying immune etiology that may be virally originating. Despite the adaptive immune response, we find evidence that T-cell immunosenescence may be associated with the rapid progression of VS. These findings provide a rationale for clinical trials evaluating immunotherapy in patients with rapidly progressing VS. Here we propose a non-platform dependent (KTSP), transcriptomic signature to allow identification of patients with rapidly progressing VS. Harnessing this signature will allow for rapid transcriptomic sequencing of surgically resected samples to stratify patients based on their risk of rapid progression, thereby identifying candidates for adjuvant multimodal therapy or immunotherapy.

## Supplementary Information

**Additional file 1.**

## Data Availability

The datasets generated and/or analysed during the current study are not publicly available to maintain individual patient privacy, but are available from the corresponding author on reasonable request.

## Abbreviations

KTSP: K-top scoring pair
VS: Vestibular schwannoma
